# Why do editors of local nursing society journals strive to have their journals included in MEDLINE?: a case study of the *Korean Journal of Women Health Nursing*

**DOI:** 10.4069/kjwhn.2023.09.11.01

**Published:** 2023-09-26

**Authors:** Sun Huh

**Affiliations:** Department of Parasitology and Institute of Medical Education, College of Medicine, Hallym University, Chuncheon, Korea

## Congratulations on the *Korean Journal of Women Health Nursing* becoming a MEDLINE journal

I discovered that the *Korean Journal of Women Health Nursing* (KJWHN) has been listed as a MEDLINE journal on the “Journals Recently Accepted for Inclusion in MEDLINE” website (https://www.nlm.nih.gov/medline/medline_new_titles.html) on August 26, 2023. This list of recently accepted journals was published on August 25, 2023 (EST). I was thrilled to see KJWHN on the list, as it is a member journal of both the Korean Council of Science Editors and the Korean Association of Medical Journal Editors, organizations where I have served as president. I extend my congratulations to the editors, society executives, and all society members for their hard work in getting the journal listed in MEDLINE—“the United States National Library of Medicine’s (US NLM) premier bibliographic database that contains more than 29 million references to journal articles in life sciences with a concentration on biomedicine.” Another important partner was the publisher, which provides services such as XML and homepage production, English proofreading, and manuscript editing. It is indeed a significant challenge for an academic society in Korea to have a journal listed in MEDLINE. KJWHN is only the third journal in the nursing category in Korea to receive this honor. This accomplishment is a testament to the tireless efforts of all society members, particularly the expertise of the editor-in-chief, Dr. Sue Kim, who has held this position since January 2020.

## Benefits of becoming a MEDLINE journal

Why do editors of local nursing society journals strive for inclusion in MEDLINE? In addition to the reasons previously cited for other journals [1], the primary advantages of being a MEDLINE journal include being searchable on PubMed and being indexed with Medical Subject Headings (MeSH) keywords. KJWHN was approved for listing in PubMed Central (PMC) on July 30, 2022. Consequently, its full text has been searchable in PMC, and its abstracts have been accessible in PubMed since the first issue of 2020 [2]. Therefore, it was already searchable in PubMed through its indexing in PMC. Now, as a MEDLINE journal, the addition of MeSH indexing is another significant benefit. Research articles indexed with MeSH keywords are more likely to be viewed by researchers worldwide than those without MeSH indexing, due to the enhanced sensitivity and specificity of search results that MeSH provides.

There are other advantages of becoming a MEDLINE journal, although KJWHN has already attained them. For instance, a third advantage is that a MEDLINE journal is searchable in the Scopus database. “For the majority of MEDLINE titles, Scopus has agreements with the publishers directly and receives the content from them. There are around 125 titles for which Scopus has permission to cover, and MEDLINE supplies directly to Scopus” [3]. The fourth advantage is exemption from the review of scientific quality during the PMC application process. Since KJWHN was already indexed in Scopus [4] and successfully passed the PMC process in 2022 [2], these two benefits are not applicable. The fifth advantage is the eligibility to add previous articles to PubMed and PMC, regardless of their language, if appropriate XML files are provided. This retrospective indexing is typically limited to the same journal title.

## Criteria of high scientific quality

In addition to the five measurable benefits of being a MEDLINE journal, the most significant advantage is the enhanced prestige of the journal brand. This is because the journal’s content undergoes rigorous evaluation by experts from the Literature Selection Technical Review Committee (LSTRC) in the same field, ensuring the scientific quality of the content. This assessment of scientific quality is independent of citation frequencies. The LSTRC evaluates five aspects: scope and coverage, editorial policies and processes, scientific rigor of article content, production and administration, and impact. From my years of observation and analysis of why many Korean biomedical journals fail the MEDLINE evaluation process, I believe that the critical concept of acceptable scientific rigor centers on the following issues:

-Is the study design stated and the main text described according to the corresponding reporting guidelines?

-Is there a hypothesis statement in an experimental or analytic study?

-Is there a sample size estimation for a study with a hypothesis statement?

-Is the statistical analysis appropriate?

-Is the background for variable selection explained?

-Is the interpretation reasonable and not exaggerated?

KJWHN passed those evaluation items successfully according to the LSTRC Journal Review Summary Report dispatched to the journal.

## Narrow path to MEDLINE coverage for nursing society journals from non-English speaking countries

**Figure 1.**
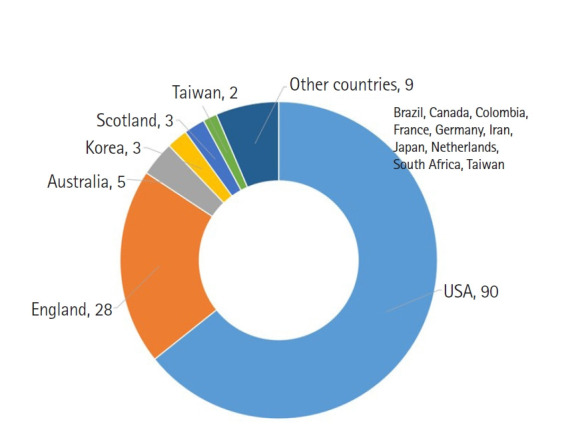
Number of MEDLINE journals in the nursing category according to country (as of August 30, 2023).

A list of nursing journals currently indexed in MEDLINE is available in Supplementary Table 1. The search term in the NLM Catalog was “currentlyindexed AND (nursing [title] OR nurse [title]).” Out of 141 results, one was removed since it was not a nursing journal. Of the remaining 140 journals, only 22 (15.76%) originate from countries other than the United States and England. With the acceptance of KJWHN, Korea now ranks fourth among countries in terms of MEDLINE journals in the nursing category, a position it shares with Scotland (Figure 1). The dominance of the United States and England may be attributed to the prevalence of large commercial publishers in these two countries. Of the 140 journals, 12 (8.6%) are published by nursing societies or associations, while the remaining 128 are published by commercial publishing companies (Supplementary Table 1). This trend is also observed in other scientific journals, as it is difficult for journals affiliated with an academic society to compete with those published by commercial publishers, primarily due to budget constraints or a lack of expertise in editing and publishing. Consequently, many society journals outsource their publishing to local publishers, a common practice in Korea. Outsourcing, however, requires a substantial budget, which can be provided by the society and/or the authors through article processing charges. Given the stringent criteria for MEDLINE indexing (https://www.nlm.nih.gov/medline/medline_statistics.html), Korean nursing society journals have started with PMC indexing [5] to ensure their discoverability in PubMed.

## Being indexed in MEDLINE and PMC

**Figure 2.**
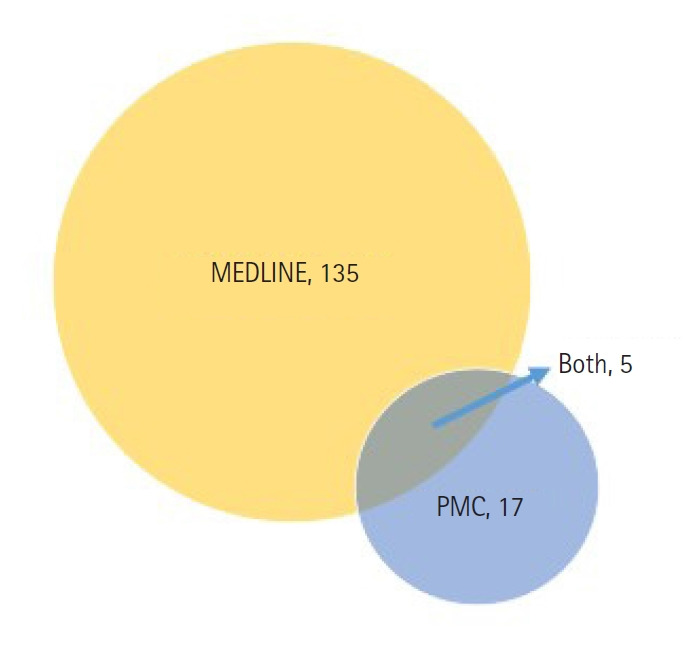
The number of nursing journals indexed in MEDLINE, PubMed Central (PMC), and both (as of August 30, 2023).

KJWHN is listed in MEDLINE and PMC simultaneously. Of the 24 nursing journals currently indexed in PMC (Supplementary Table 2) by the search term “journalspmc AND (nursing [title] OR nurse [title])” two should be removed: one journal’s title was changed, and the other entry corresponds to the proceedings from a single event. Based on this list, another Korea-based journal, *Child Health Nursing Research*, has also been indexed in PMC [5], but it has yet to be included in MEDLINE. Thus, out of the 22 PMC journals and 140 MEDLINE journals, the following five are indexed in both databases: *Investigación y educación en enfermerías*, published in Colombia; *International Journal of Community based Nursing and Midwifery*, published in Iran; *Korean Journal of Women Health Nursing*, published in Korea; *Curationis*, published in South Africa; and *Nursing Open*, published in the United States (Figure 2). MEDLINE journals are exempted from the evaluation of scientific quality for PMC indexing; therefore, providing full-text PMC XML is the only remaining step. This raises a question: why don’t the remaining 135 MEDLINE nursing journals deposit their full-text PMC XML files to PMC? Of the 140 MEDLINE nursing journals, 128 are published by commercial publishing companies, which typically do not support open access policies. The exception to this is *Nursing Open*, which has deposited its full-text PMC XML files to PMC. Among the eight MEDLINE, non-PMC journals not published by commercial publishing companies, two are open access and therefore eligible for deposit to PMC: *Revista da Escola de Enfermagem da U S P*. in Brazil and *Journal of Korean Academy of Nursing in Korea*. The decision to deposit full-text XML files to PMC ultimately lies with the publisher. The remaining six journals offer free access (1), require a subscription (4), or do not provide full text on the journal’s website (1) (Supplementary Table 1).

## What should local nursing society journals do to be eligible for MEDLINE?

Numerous information technologies have already been introduced to scholarly journal publishing. Standard journal publishing techniques or requirements include a secure URL address for the journal homepage, Journal Article Tag Suite XML [6], digital object identifiers [7], the cited-by function, Crossmark, Metrics, and a manuscript management system. Adopting these technologies or platforms is essential for survival in the journal market. However, beyond these technologies, the content of the article remains paramount. As previously mentioned, the editor must verify if the journal meets the criteria for evaluation by the LSTRC. Among the five evaluation topics, scientific rigor presents the greatest challenge. Therefore, it is crucial to adopt the appropriate study design and corresponding reporting guidelines to present the results in a logical and lucid manner. Providing an algorithm for the study design can be helpful [8,9], as well as clearly stating the appropriate study design and the corresponding reporting guidelines for a journal [10].

## Further work to maintain listing in MEDLINE

To consistently meet the scientific quality standards set by the US NLM, it is crucial that a journal adheres to the industry’s best practices. Being listed in MEDLINE is the initial step towards elevating the journal to an internationally top-tier level. The following recommendations are proposed: first, uphold ethical standards under a distinct “Ethics statement” heading; and second, ensure scientific rigor by clearly defining the study design and corresponding reporting guidelines. Like many other local society journals, KJWHN required assistance and thus hired full-time staff to work for the journal. Since all editorial work is voluntary, editor burnout may occur due to the heavy load of reviewing, editing, and administration. One potential solution to this issue could be to increase the article processing charge on the author’s side, which would make it possible to hire professional staff. It is my hope that editors will be able to continue their work without experiencing burnout and find joy and satisfaction in their voluntary roles.

## Supplementary materials

Further details on supplementary materials are presented online (available at https://doi.org/10.4069/kjwhn.2023.09.11.01).

Supplementary Table 1. MEDLINE journals in the nursing category [Cited 2023 Sep 6]

Supplementary Table 2. PubMed Central journals indexed in the nursing category [Cited 2023 Sep 6]
